# Guard-Cell Hexokinase Increases Water-Use Efficiency Under Normal and Drought Conditions

**DOI:** 10.3389/fpls.2019.01499

**Published:** 2019-11-19

**Authors:** Gilor Kelly, Aiman Egbaria, Belal Khamaisi, Nitsan Lugassi, Ziv Attia, Menachem Moshelion, David Granot

**Affiliations:** ^1^Institute of Plant Sciences, Agricultural Research Organization, The Volcani Center, Rishon LeZion, Israel; ^2^The Robert H. Smith Faculty of Agriculture, Food and Environment, The Institute of Plant Sciences and Genetics in Agriculture, The Hebrew University of Jerusalem, Rehovot, Israel

**Keywords:** guard cells, hexokinase, water-use efficiency, photosynthesis, transpiration

## Abstract

Water is a limiting resource for many land plants. Most of the water taken up by plants is lost to the atmosphere through the stomata, which are adjustable pores on the leaf surface that allow for gas exchange between the plant and the atmosphere. Modulating stomatal activity might be an effective way to reduce plants’ water consumption and enhance their productivity under normal, as well as water-limiting conditions. Our recent discovery of stomatal regulation by sugars that is mediated by guard-cell hexokinase (HXK), a sugar-sensing enzyme, has raised the possibility that HXK might be used to increase plant water-use efficiency (WUE; i.e., carbon gain per unit of water). We show here that transgenic tomato and *Arabidopsis* plants with increased expression of HXK in their guard cells (GCHXK plants) exhibit reduced transpiration and higher WUE without any negative effects on growth under normal conditions, as well as drought avoidance and improved photosynthesis and growth under limited-water conditions. Our results demonstrate that exclusive expression of HXK in guard cells is an effective tool for improving WUE, and plant performance under drought.

## Introduction

Agriculture accounts for most of the world’s consumption of fresh water and, in many plant species, more than 98% of the water taken up by the roots is lost to the atmosphere *via* the stomata (Morison et al., 2008). For this reason, efforts have been made to improve stomatal behavior to reduce water loss. Stomata are dynamic pores in the impermeable protective cuticle that coats the aerial parts of land plants. Each stoma is comprised of two guard cells and the pore they circumscribe. In most crops, stomata open in response to light, to allow the entry of atmospheric carbon dioxide (CO_2_) for photosynthesis, but that happens at the cost of extensive water loss through the open stomata. The ratio between carbon (biomass) gain *via* photosynthesis and water loss *via* transpiration is termed water-use efficiency (WUE). In C3 plants, WUE is low and for every CO_2_ molecule fixed in the Calvin cycle, about 500 molecules of water are transpired to the atmosphere. Stomata close in response to various environmental and physiological conditions such as dark, a low photosynthesis rate and water stress, in order to reduce water loss. Various approaches for reducing transpiration and increasing WUE have been tested. These approaches include enhanced production of abscisic acid, a stress hormone that closes stomata (Thompson et al., 2007); modifying abscisic acid perception (Park et al., 2015); decreasing the number of stomata per unit of leaf area (Yu et al., 2008; Franks et al., 2015; Hughes et al., 2017) and modulating metabolic and osmotic pathways that affect stomatal aperture (Antunes et al., 2012; Antunes et al., 2017). While some of these approaches do increase WUE, some have negative effects on photosynthesis, biomass production and yield (Thompson et al., 2007; Yoo et al., 2009; Park et al., 2015; Flexas, 2016; Antunes et al., 2017).

It was previously shown that sugars stimulate stomatal closure (Kelly et al., 2013). During the day, sugar production may exceed a plant’s ability to translocate and use that sugar, so that sugar accumulates in leaves (Ewert et al., 2000; Outlaw and De Vlieghere-He, 2001; Kang et al., 2007). Some sugar is carried by the transpiration stream toward the guard cells (Lu et al., 1997; Ewert et al., 2000; Outlaw and De Vlieghere-He, 2001; Kang et al., 2007), enters the guard cells and is sensed by hexokinase (HXK), a sugar-sensing enzyme that stimulates stomatal closure (Kelly et al., 2013; Li et al., 2016; Hei et al., 2017; Medeiros et al., 2018). Since sugar production happens at the expense of significant water loss, stomatal closure by sugars forms a feedback mechanism that coordinates the production and use of sugar with water loss (Kelly et al., 2013; Granot et al., 2014; Lawson et al., 2014; Granot and Kelly, 2019).

In support of the role of HXK in guard cells, increased expression of *Arabidopsis* HXK (*AtHXK1*) specifically in guard cells of *Arabidopsis*, tomato (*Solanum lycopersicum*) and citrus [Troyer citrange (*Citrus sinensis* x *Poncirus trifoliata*)] plants, driven by the guard cell-specific promoter KST1 (Kelly et al., 2017a), was shown to reduce stomatal apertures and decrease transpiration by about 20% without any noticeable negative effects on growth. Plants with lower transpiration rates that grow normally are ideal candidates for improving whole-plant WUE and reducing irrigation requirements. Yet, to date, the water-use characteristics of these plants and their performance under limited-water-supply conditions have not been examined. In this study, we thoroughly analyzed the WUE of GCHXK plants using gas-exchange tools, as well as a precise lysimeter system. We show that WUE is significantly higher in tomato and *Arabidopsis* plants expressing HXK in their guard cells, under normal growth conditions. In addition, these plants exhibit drought avoidance and improved photosynthesis and growth under limited-water conditions. Our data demonstrate the potential of the GCHXK method for improving plant performance, which may be exploited in other crop species.

## Materials and Methods

### Plant Material and Growth Conditions

Experiments were conducted using WT tomato (*S. lycopersicum*, cv. MP-1) plants and independent isogenic transgenic homozygous tomato lines expressing *AtHXK1* under the control of the *KST1* promoter (GCHXK lines), as well as WT *Arabidopsis* (Col.) and two transgenic homozygous *Arabidopsis* lines expressing *AtHXK1* under the control of the *KST1* promoter (Kelly et al., 2013). The tomato plants were grown in a greenhouse in which temperature was partially controlled, with midday temperatures of 25 to 30°C, under natural sunlight and ambient growth conditions. The *Arabidopsis* plants were grown in a walk-in growth chamber kept at 22°C, with a 10-h light/14-h dark (short-day) photoperiod.

### Gas-Exchange Measurements

Gas exchange was measured using the LI-6800 portable gas-exchange system (LI-COR, Lincoln, NE, USA). The tomato analyses were performed on fully expanded leaves (5^th^–6^th^ leaf from top) under favorable growth conditions. Photosynthesis was induced under saturating light (700 µmol m^-2^ s^-1^) with 400 µmol mol^-1^ CO_2_ surrounding the leaf (*C*_a_) and 10% blue light of photosynthetically active photon flux density. The flow rate was set to 500 µmol air s^-1^. The leaf-to-air vapor pressure deficit was kept around 0.9–1 kPa during the measurement. Leaf temperature was ∼28°C (ambient greenhouse temperature). Measurements were performed between 10:00 a.m. and 1:00 p.m. The gas-exchange analysis of *Arabidopsis* was conducted as described by Flexas et al. (2007) and Sade et al. (2014). Photosynthesis was induced under light (1,000 µmol m^-2^ s^-1^ for the work described in **Figure 5** and 200 µmol m^-2^ s^-1^ for the work described in **Figure 6**) with 400 µmol mol^-1^ CO_2_ surrounding the leaf (*C*_a_). The amount of blue light was set to 10% of the photosynthetically active photon flux density. The flow rate was set to 150 µmol air s^-1^, and the leaf-to-air vapor pressure deficit was kept around 1–1.1 kPa during the measurement Leaf temperature was 22°C. Mesophyll conductance of CO_2_ (*g*_m_) was assayed as described in detail by Flexas et al. (2007); Kelly et al. (2014) and Sade et al. (2014). For the *A*_N_/*C*_i_ and *g*_S_/*C*_i_ curves, light intensity was kept constant (1,000 µmol m^-2^ s^-1^) over the course of the measurements and each leaf was first adapted to 400 ppm CO_2_. *C*_a_ was then decreased in a step-wise manner (400, 300, 200, 100, 50 ppm) and then increased to 600, 800, 1,000, 1,200, or 1,400 ppm. Photosynthesis and stomatal conductance were measured at each *C*_a_ point. There were five independent biological repeats for the WT plants and for the GCHXK plants and the experiment was repeated two times with similar results. Measurements were performed between 10:00 a.m. (3 h after the light was switched on) and 2:00 p.m.

### Whole-Plant Transpiration, Continuous Stomatal Conductance of the Whole Canopy and Water-Use Efficiency Measured Using the Lysimeter Method

Whole-plant relative daily transpiration (RDT) was determined using lysimeters, as described in detail by Halperin et al. (2016). Individual WT and GCHXK7 and GCHXK12 transgenic tomato plants were planted in 3.9-l pots and grown under controlled conditions. Each pot was placed on a temperature-compensated load cell with a digital output and was sealed to prevent any evaporation from the surface of the growth medium. A wet vertical wick partially submerged in a 1-l water tank was placed on a similar load cell and used as a reference for the temporal variations in the potential transpiration rate. The output of the load cells was monitored every 10 s and the average readings over 3-min periods were collected in a data-logger for further analysis. Whole-plant transpiration was calculated as a numerical derivative of the load cell output following a data-smoothing process (Sade et al., 2010). Each plant’s daily transpiration rate was normalized to its weight and/or its total leaf area (measured using the LI-3100 area meter, LI-COR) and the data for the nearby submerged wick and these figures were averaged for a given line over all plants (amount taken up by the wick daily = 100%), as described in detail by Halperin et al. (2016). For the drought experiments, irrigation was fully stopped for several days, as detailed in each figure. When indicated, a recovery stage in which irrigation was resumed was included. Continuous stomatal conductance of the entire canopy [*gs*_c _(mmol s-1 m-2)] was calculated by dividing the whole-plant transpiration rate, *E*, by the vapor pressure difference (VPD) using Equation 1, in which *P*_atm_ is the atmospheric pressure (101.3 kPa).

(1) $$gs_{\text{c}}=(E\cdot P_{\mathrm{atm}})/\mathrm{VPD}$$

VPD (Equation 2) is the difference (in kilopascal) between the vapor pressure of the saturated air and the vapor pressure of the ambient air. In plants, this refers to the difference between the pressure in the substomatal cavities and the atmospheric pressure. In Equation 2, *T* is the air temperature (degrees Celsius), RH is the relative humidity (0–1), 0.611 is the saturation vapor pressure at 0°C and 17.502 and 240.97 are constants (Buck, 1981). The temperature and RH in the greenhouse were monitored using sensors (HC2-S3-L; Rotronic, Bassersdorf, Switzerland).

(2) $$VPD=(1-RH)0.611e^{\left(\frac{17.502+T}{240.97+T}\right)}$$

The *gs*_c_, *E*, total water loss and RDT between 11:00 a.m. and 1:00 p.m. were averaged for each plant and then averaged between all plants of a particular line. Plant weight was measured each day and a factor linking plant weight to leaf area was included in the calculations and verified throughout the experiment by assaying plant weight and leaf area of plants neighboring the experimented plants, which were exposed to the same growth conditions.

### Water-Use Efficiency Calculations

WUE was determined by measuring gas exchange (tomato and *Arabidopsis*) and by the lysimeter system (tomato). For the gas-exchange analyses, instantaneous water-use efficiency (*I*WUE) was calculated by dividing *A*_N_ by *T*_r_, as described by Sade et al. (2010). For the lysimeter analyses, WUE was calculated as the weight added each day, divided by the total water loss from each plant (daily plant weight accumulation/daily water loss). Values were then averaged for all plants in each line.

### *Arabidopsis* Drought Assay

For the *Arabidopsis* drought experiment, seeds were germinated in pots that each contained 110 g of soil. Two weeks after germination, the seedlings were thinned to one seedling per pot. Eight-week-old plants were exposed to a gradual increase in drought conditions by stopping the irrigation. Gas-exchange analysis was determined at four time points over the course of the experiment (days 5, 8, 12, and 16) as the drought intensified. Volumetric soil water content (SWC) was measured using the ProCheck device connected to an EC-5 soil probe (Decagon Devices, Pullman, WA, USA). An average of three reads were taken per pot and the data were averaged over all of the pots per line, for each time point. SWC was assayed parallel to gas-exchange measurements, to estimate drought severity. This experiment was repeated twice with similar results.

For the work described in **Figures S1** and **S2**, four plants were left in each pot after thinning, in order to induce a faster drought response. For the work described in **Figure S1**, at the end of the drought phase, which was achieved by withholding water for 13 days, the whole rosette was cut above the ground and imaged. For the work described in **Figure S2**, three mild drought cycles were applied. Plants were subjected to drought until dry soil was visible and were then watered, followed by second and third drought cycles and additional re-watering. At the end of the third drought cycle, the whole rosette was cut above the ground and taken for leaf-area analysis. Leaf area was determined using the ImageJ software’s (http://rsb.info.nih.gov/ij/) wand (tracing) tool.

### RNA Extraction, Complementary DNA Preparation, and Quantitative Real-Time Polymerase Chain Reaction

RNA extraction, complementary DNA preparation and quantitative real-time PCR analysis were performed precisely as described by Lugassi et al. (2015). Data were normalized using the tomato *SlCyP* (cyclophilin—accession; M55019) as a reference gene. The primers used for amplification were: *AtHXK1* (Fwd-AAACCTACCCAAAGAGCGCC, Rev-TGACGCCTTAGAACTTGGCT) and *SlCyP* (Fwd-CGTCGTGTTTGGACAAGTTG, Rev-CCGCAGTCAGCAATAACCA).

### Statistical Analysis

Statistical analysis was performed using JMP 5.0 software. Means were compared using Student’s *t*-test or Dunnett’s test (compared with control—WT), as specified in the description for each experiment. Means were considered to be significantly different at *P* < 0.05.

## Results

### Elevated Expression of HXK in Tomato Guard Cells Reduces Transpiration and Increases Water-Use Efficiency Under Normal and Drought Conditions

**Figure 1:**
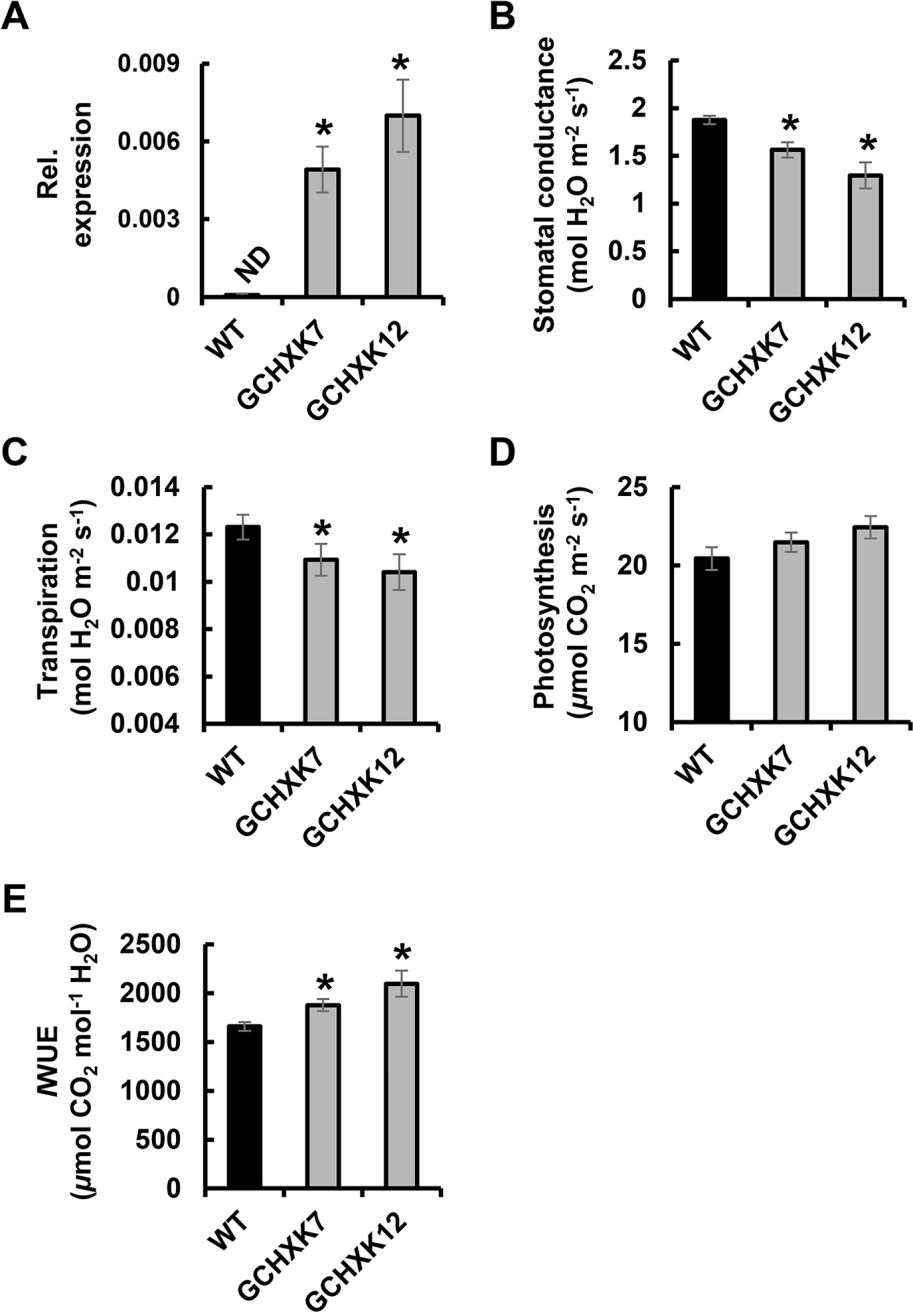
Gas-exchange and expression data of GCHXK tomato plants. **(A)** Relative expression of *AtHXK1* was examined using RNA extracted from mature leaves of wild-type (WT, black columns) and GCHXK lines (GCHXK7 and GCHXK12, gray columns). *SlCyP* was used for normalization. **(B)** Stomatal conductance, **(C)** transpiration, and **(D)** photosynthesis of WT and GCHXK lines. **(E)** Instantaneous water-use efficiency (*I*WUE) calculated by dividing *A*_N_ by *T*_r_. Data are means of seven **(**for **A)** and eight **(**for **B**–**E)** independent biological replicates ± SE. An asterisk denotes a significant difference relative to the WT (Dunnett’s test, *P* < 0.05).

The WUE of tomato plants expressing *AtHXK1* specifically in their guard cells (GCHXK plants) was assayed using two methods; (1) gas-exchange analysis and (2) a lysimeter-scale system that allows for continuous measurement of water loss and biomass gain. We examined two independent lines, GCHXK7&12, in which the expression of *AtHXK1* was verified by real-time polymerase chain reaction (**Figure 1A**). Gas-exchange analysis of GCHXK tomato lines grown under favorable growth conditions revealed reduced stomatal conductance (*g*_s_) and transpiration (*T*_r_) with no reduction in the rate of photosynthesis (*A*_N_), that is, an increase in the overall instantaneous water-use efficiency (*I*WUE; **Figures 1B–E**), calculated as the rate of photosynthesis divided by the transpiration rate.

**Figure 2:**
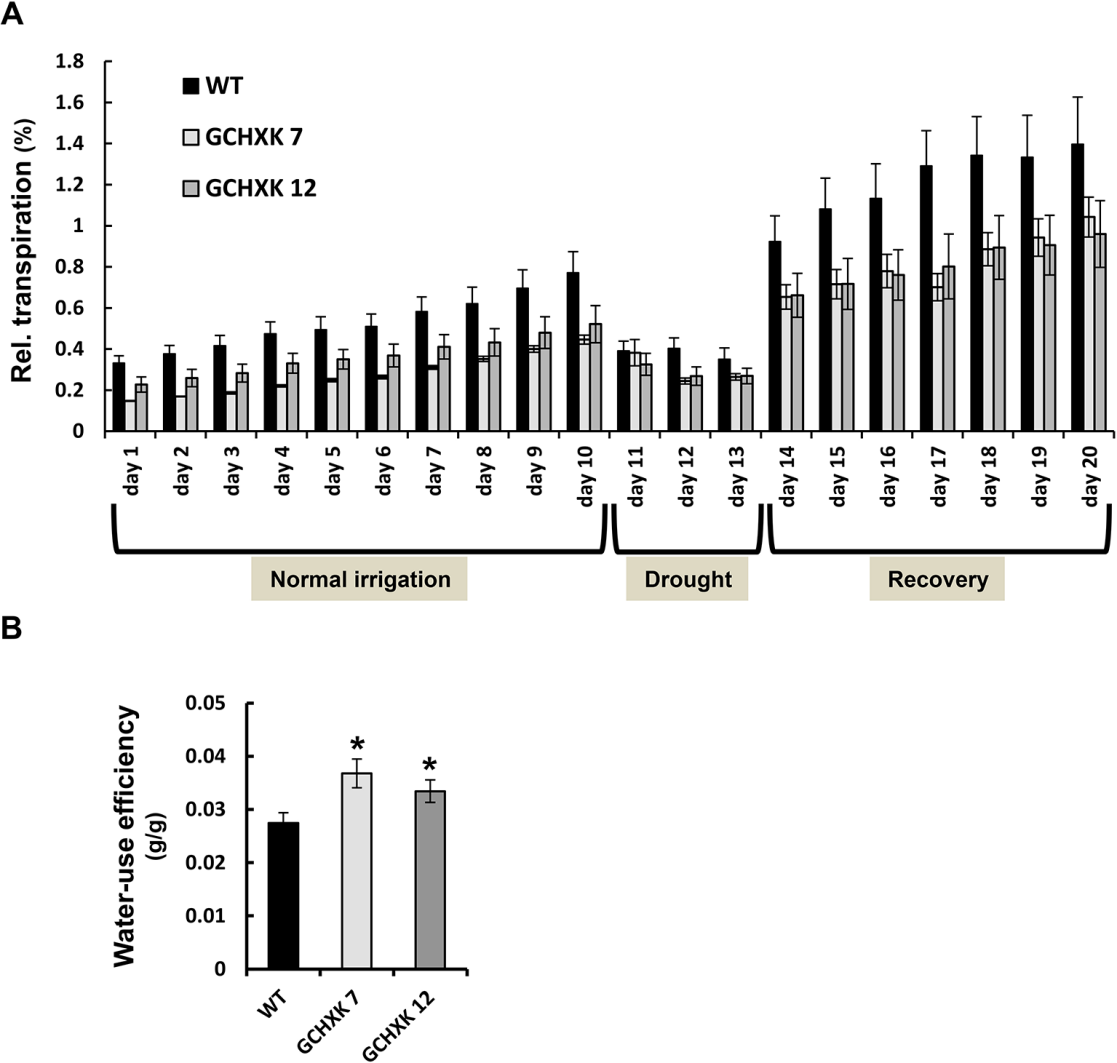
Transpiration and WUE of GCHXK tomato plants as determined using the lysimeter system. Whole-plant relative daily transpiration of WT tomato plants and two independent transgenic GCHXK lines (GCHXK7, GCHXK12). Plants were grown under conditions of normal irrigation for 10 days and then subjected to drought stress for 3 days, followed by recovery irrigation for an additional 7 days. **(A)** Day-by-day relative daily transpiration over the course of the experiment. Data were normalized to total plant weight. **(B)** WUE was calculated as the ratio between plant weight accumulation and water loss for each plant during the normal-irrigation stage. Data are means of five independent biological replicates ± SE. When not seen, SE is smaller than the column border; an asterisk indicates a significant difference relative to the WT (Dunnett’s test, *P* < 0.05).

To examine the whole-plant diurnal behavior of GCHXK7&12 plants, we performed continuous measurements of the transpiration and daily weight gain of each individual plant, using a precise and sensitive lysimeter-scale system (Halperin et al., 2016) (**Figure 2**). Eight-week-old plants were monitored under normal irrigation conditions for 10 days and were then subjected to a gradual increase of drought stress imposed by stopping irrigation for 3 days, followed by recovery irrigation for additional 7 days. The RDT of the GCHXK plants was lower than that of the WT plants throughout the entire experiment (**Figure 2A**). The continuous measurements allowed us to calculate WUE as cumulative weight gain per total water loss for each plant. The mean WUE was more than 20% higher in the GCHXK lines than among the WT plants (**Figure 2B**). Stopping irrigation after 10 days resulted in a steep drop (∼50%) in the transpiration of WT plants within 1 day (day 11, **Figure 2A**). In contrast, the transpiration of the GCHXK plants was affected only slightly (∼25%) on the first day (day 11) and by about 40% on days 12 and 13. This may indicate that GCHXK plants are less sensitive to water limitation, most likely due to their consistently lower transpiration rates, which allow for drought avoidance (i.e., gradual usage and preservation of soil water).

### Transpiration of GCHXK Plants Over a Longer Drought Period

**Figure 3:**
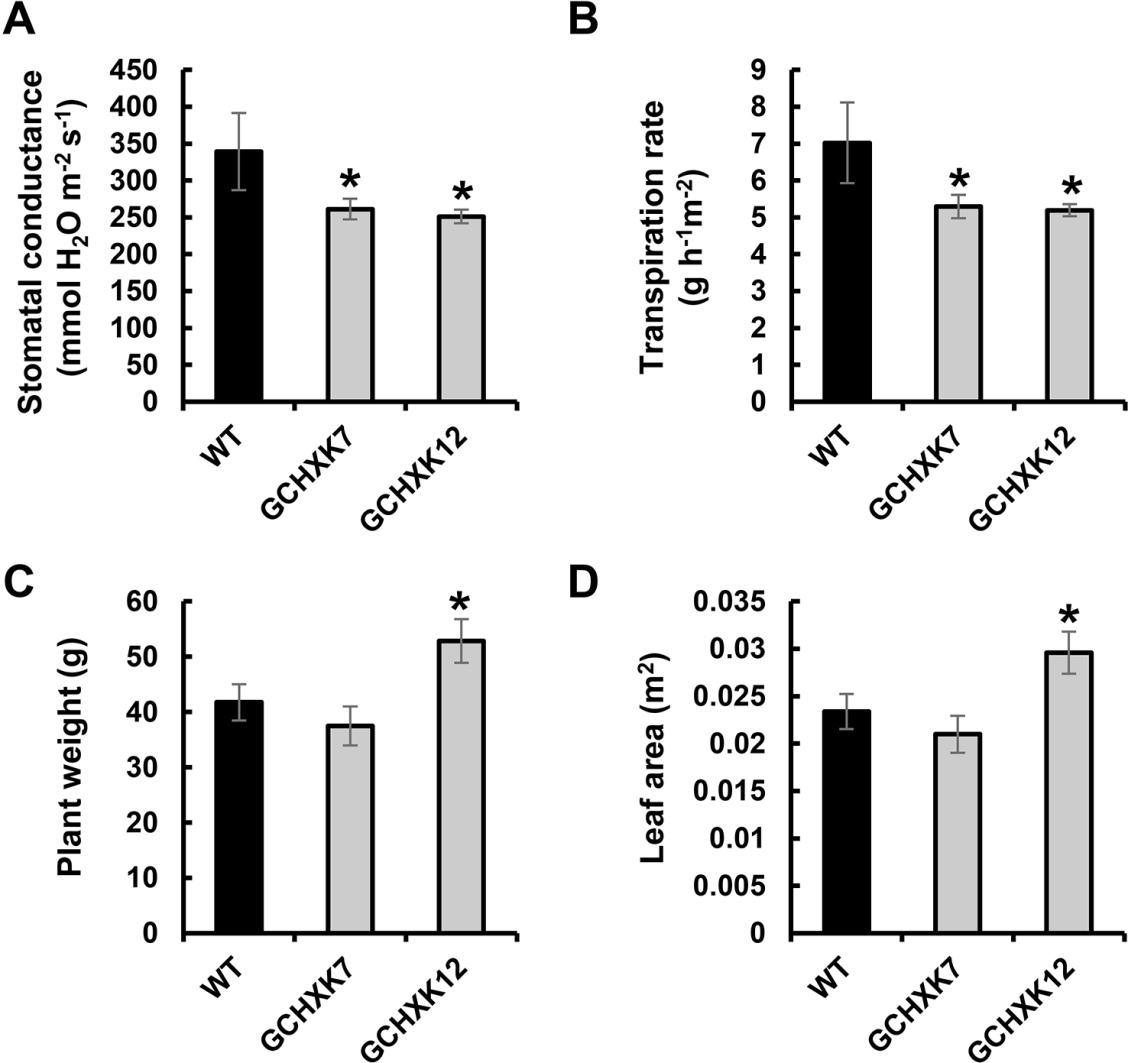
Transpiration, stomatal conductance and biomass accumulation of GCHXK tomato plants as determined using the lysimeter system. **(A)** Whole-canopy stomatal conductance, **(B)** transpiration rate, **(C)** total plant fresh weight, and **(D)** total leaf area of wild-type (WT, black columns) and two independent transgenic GCHXK lines (GCHXK7, GCHXK12, gray columns) under well-watered conditions. **(A**–**D)** Data are means of eight independent biological replicates ± SE. Asterisks denote significant differences relative to the WT (Dunnett’s test, *P* < 0.05).

To examine the behavior of GCHXK plants over a longer drought period, the lysimeter experiment was repeated using younger (6-week-old) plants, so that the soil water content would decrease more gradually. This experiment included measurements of leaf area, in addition to measurements of plant weight (**Figure 3**). In agreement with the results of the gas-exchange analysis presented in **Figure 1**, the transpiration and stomatal conductance of the GCHXK plants under well-watered conditions (measured by the lysimeter system and averaged between 11:00 a.m. and 1:00 p.m.) were significantly lower than those of the WT plants (**Figures 3A, B**). Despite this reduced stomatal conductance, plant weight and total leaf area were not negatively affected in the GCHXK lines (**Figures 3C, D**).

**Figure 4:**
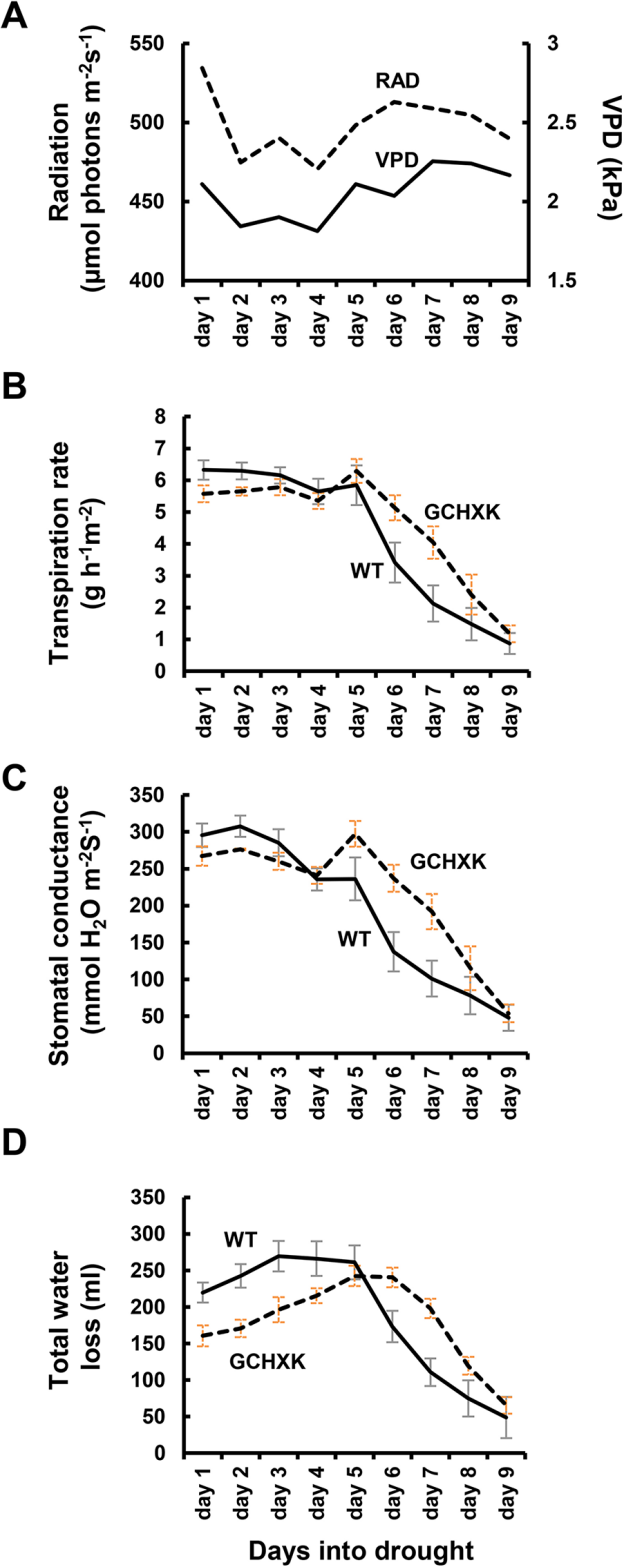
GCHXK tomato plants under intensifying drought conditions. WT plants (solid line, SE bars in gray) and GCHXK plants (dashed line, SE bars in orange) were exposed to increasing drought conditions for 9 days by stopping the irrigation. **(A)** VPD and radiation were measured each day during the experiment. **(B)** Transpiration rate, **(C)** stomatal conductance, and **(D)** total water loss (not normalized; data for WT and GCHXK were averaged each day at midday, for 9 days over the drought period). **(B**–**C)** Data were normalized to leaf area. Data are presented as means of eight independent biological replicates ± SE.

The plants were then exposed to a gradual increase in drought stress due to irrigation cessation and their transpiration rate, stomatal conductance and total water loss (the absolute amount of water transpired) were measured for nine consecutive days (**Figure 4**). During the first 3 days following the cessation of irrigation, the stomatal conductance and transpiration rates of WT plants decreased slightly, while the stomatal conductance and transpiration rates of the GCHXK, which were already lower than those of the WT plants, were not affected (**Figures 4B, C**). On the fourth and fifth days, the transpiration rates of the WT and GCHXK were similar and the transpiration rate of GCHXK even increased on the fifth day due to an increase in VPD (**Figures 4A, B**). On the sixth, seventh and eighth days, the transpiration rates and stomatal conductance of both the WT plants and the GCHXK plants decreased despite the high VPD, indicating a notable water deficit. However, from day 6 on, the GCHXK plants maintained higher transpiration rates than the WT, peaking at an almost 2-fold higher rate on day 7 (**Figure 4B**). Finally, on the ninth day, the transpiration rate of the GCHXK plants fell to the level of that of the WT plants (**Figure 4B**, day 9). These results, together with the results presented in **Figure 2**, indicate that at the beginning of the drought period, GCHXK plants were less affected, and as drought proceeded, the transpiration rate and stomatal conductance declined sharply in the WT and only moderately in the GCHXK plants (**Figure 4B, C**). As a result, the total water loss of GCHXK plants per day was clearly lower than that of the WT plants during the first 5 days of drought (e.g., 196 ml for GCHXK compared to 269 ml for WT at day 3; **Figure 4D**). That inevitably led to soil water preservation that allowed GCHXK plants to keep their stomata open and maintain higher transpiration rates at later stages (e.g., 240 ml for GCHXK as compared to 173 ml for the WT at day 6; **Figure 4D**). Together, these results demonstrate that the GCHXK plants exhibited more efficient water management, consumed less water, and were less exposed to water limitation throughout the drought period.

### GCHXK and Water-Use Efficiency of *Arabidopsis*

**Figure 5:**
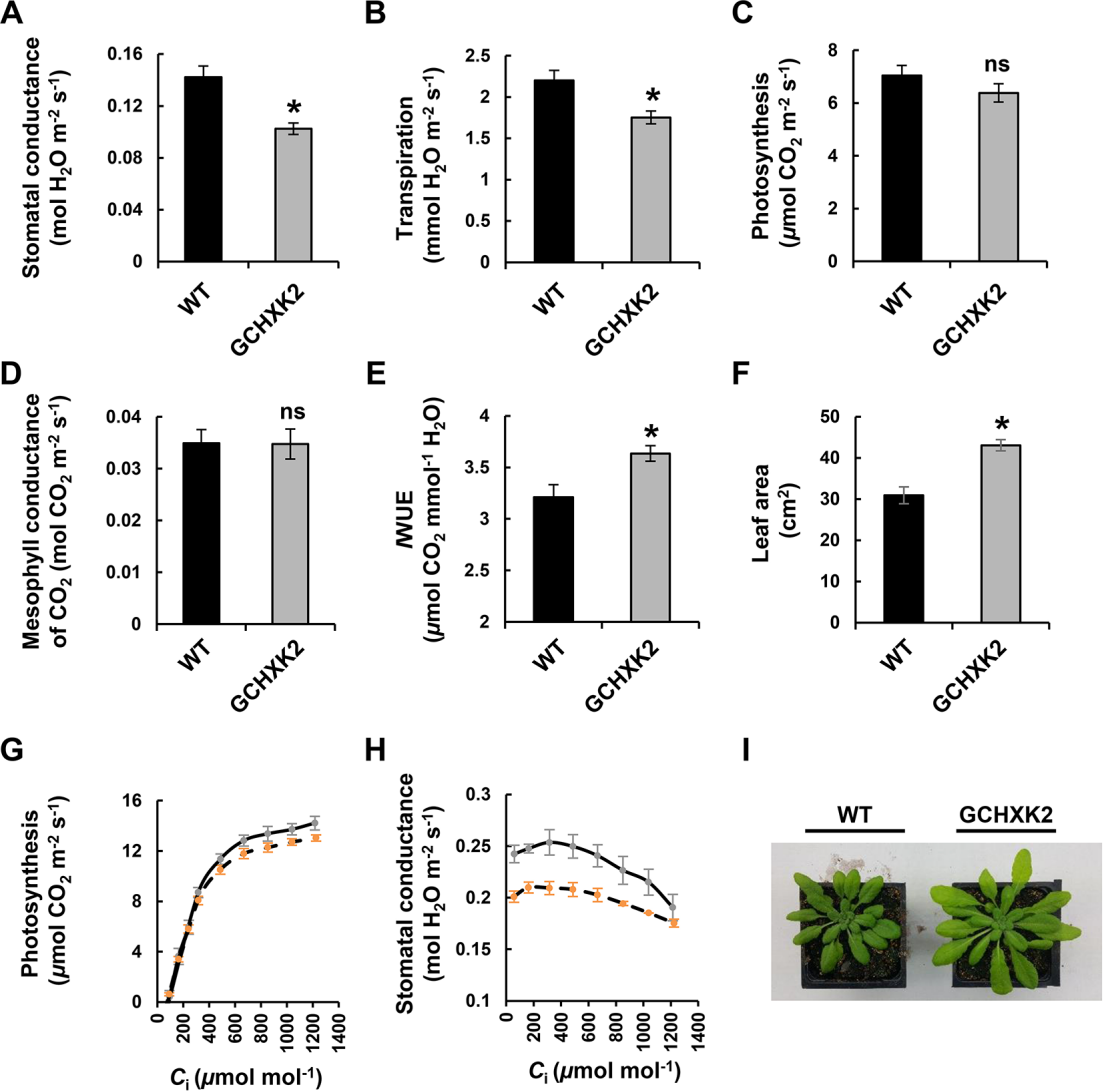
Gas exchange of GCHXK *Arabidopsis* plants. **(A)** Stomatal conductance, **(B)** transpiration, **(C)** photosynthesis, and **(D)** mesophyll conductance of CO_2_ in WT (black columns) and GCHXK2 plants (gray columns). **(E)** Instantaneous water-use efficiency (*I*WUE) calculated by dividing *A*_N_ by *T*_r_. **(F)** Total leaf area of GCHXK2 as compared to the WT. **(G)** *A*_N_/*C*_i_ and **(H)** *g*_S_/*C*_i_ curves for the WT (solid line, SE bars in gray) and GCHXK2 (dashed line, SE bars in orange). **(I)** Representative images of 35-day-old WT and GCHXK2 plants. **(A**–**E**, **G**, **H)** Measurements were performed between 10:00 a.m. and 2:00 p.m. using mature plants (50 to 60 days old), Data points for **A**–**F** are means of eight (for A–E) and seven (for F) independent biological replicates ± SE. An asterisk denotes a significant difference relative to the WT (*t*-test, *P* < 0.05); ns, not significant. Data points for **(G** and **H)** are means of five independent biological replicates ± SE.

Gas-exchange analysis was used to study the effect of GCHXK on WUE in *Arabidopsis* as well (**Figure 5**). Stomatal conductance and transpiration were significantly reduced in GCHXK *Arabidopsis* plants (**Figures 5A, B**, respectively). Nevertheless, the rate of photosynthesis (**Figure 5C**) and the mesophyll conductance of CO_2_ (**Figure 5D**) were not affected and, as a result, the *I*WUE of the GCHXK *Arabidopsis* plants was about 13% greater than that of the control *Arabidopsis* plants (**Figure 5E**). In addition, the growth of the GCHXK plants was not impaired by the reduction in stomatal aperture. On the contrary, GCHXK leaf area was even greater than that of the WT (**Figures 5F, I**), demonstrating that, in the GCHXK plants, growth promotion coincided with high *I*WUE. A/*C*_i_ and *g*_s_/*C*_i_ data also indicated that the WUE of GCHXK was improved. Photosynthesis of GCHXK was similar to that of the WT at *C*_i_ concentrations up to 400 ppm and was slightly lower than that of the WT at higher concentrations of *C*_i_ (**Figure 5G**). The stomatal conductance (*g*_s_) of the GCHXK plants was significantly lower than that of the WT at all of the *C*_i_ concentrations, except for 1,200 ppm (**Figure 5H**). This demonstrates that the WUE of GCHXK is greater than that of the WT.

**Figure 6:**
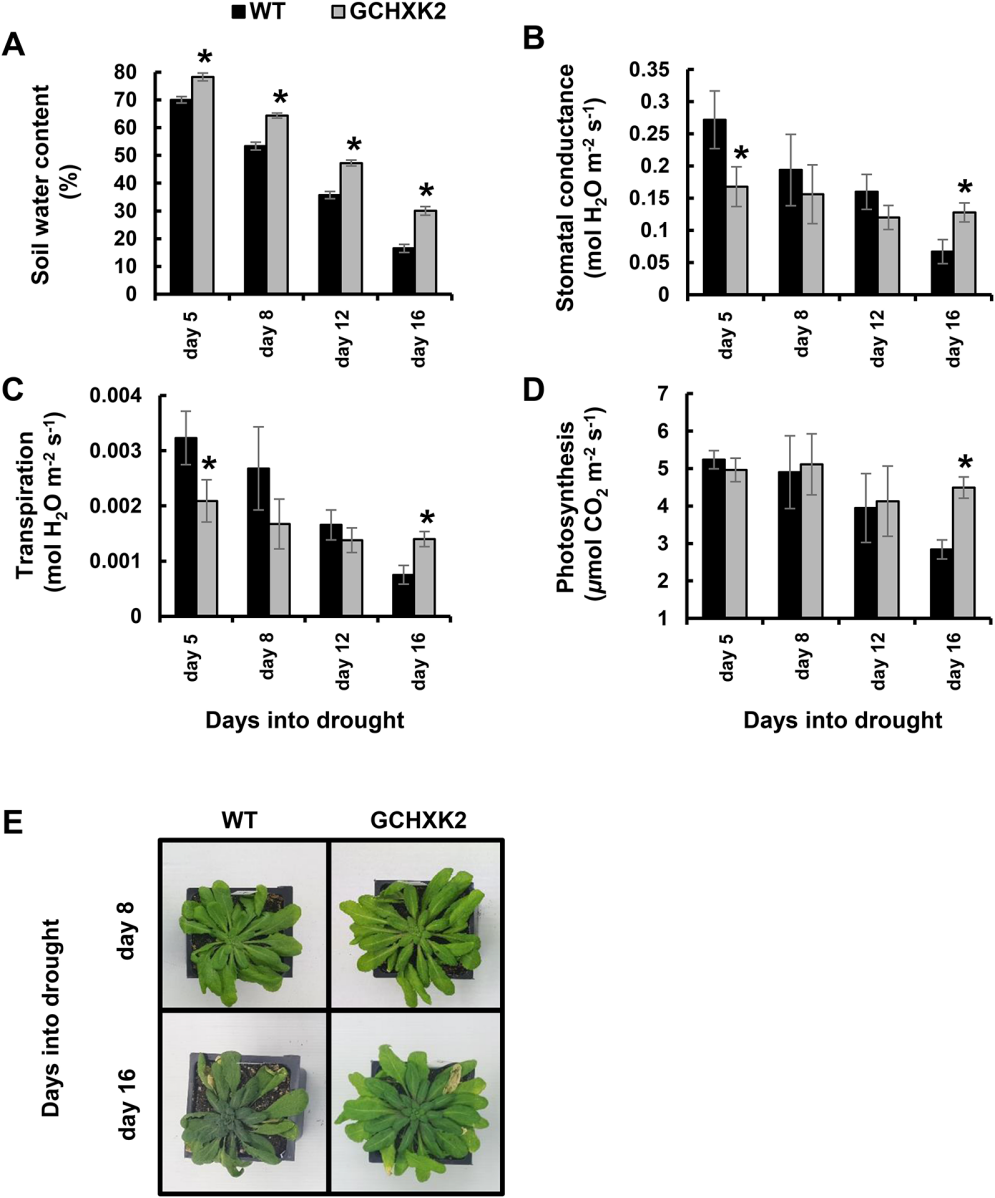
Performance of *Arabidopsis* GCHXK plants under intensifying drought conditions. Eight-week-old WT (black columns) and GCHXK2 plants (gray columns) were grown under intensifying drought conditions for 16 days. **(A)** Volumetric soil water content, **(B)** stomatal conductance, **(C)** transpiration, and **(D)** photosynthesis were measured at days 5, 8, 12, and 16 of the drought. **(B**–**C)** Data points are presented as means of 6–9 independent biological replicates ± SE. An asterisk denotes a significant difference relative to the WT for each day (*t*-test, *P* < 0.05). **(E)** Representative images of WT and GCHXK plants at days 8 and 16, prior and subsequent to severe drought stress, respectively.

Next, we exposed 8-week-old *Arabidopsis* plants (WT and GCHXK2) to a gradual increase in drought stress for 16 consecutive days, by fully stopping the irrigation (**Figure 6**). Gas exchange and soil water content (SWC) were measured on selected days during the drought period. The SWC of GCHXK was significantly higher throughout the entire drought experiment, demonstrating that the soil drying of the GCHXK plants is delayed relative to that of the WT (**Figure 6A**). On the fifth day of the drought treatment, the transpiration and stomatal conductance of the GCHXK plants were significantly lower than those of the WT plants, while their photosynthesis levels were similar (**Figures 6B–D**). These results are similar to the behavior of well-watered GCHXK plants (**Figure 5**), indicating that, at that point, the drought treatment had not yet had an effect. At later stages of drought (days 8, 12, and 16), steep reductions in transpiration and stomatal conductance were observed for the WT, as compared to the more moderate reductions observed for the GCHXK (**Figures 6B, C**). At day 16, deep into the drought period, the transpiration and stomatal conductance of WT plants were reduced by ∼76% relative to day 5 (**Figures 6B, C**), while the transpiration and stomatal conductance of the GCHXK plants were reduced by only ∼33%, displaying significantly higher levels of transpiration and stomatal conductance and significantly higher photosynthesis rates (**Figures 6B–D**, day 16).

These results are in line with the behavior observed for tomato GCHXK plants (**Figure 4**), showing that at the beginning of the drought period, GCHXK plants were less sensitive to the effect of drought, due to lower transpiration, leaving more water in the soil (**Figures 6A, C**). This drought avoidance strategy employed by the GCHXK plants helped to maintain rather high productivity (as seen by the higher levels of photosynthesis; day 16 in **Figure 6D**) at stages at which WT plants were wilting (day 16, **Figure 6E**). It appears then, that in *Arabidopsis*, as in tomato a more efficient strategy of water management is adapted by the GCHXK plants, specifically, lower water consumption under normal conditions, which entails less exposure to soil water deficit at early stages of drought (**Figures 4** and **6**).

## Discussion

### GCHXK, Water-Use Efficiency and High Photosynthesis Rates

Expressing HXK specifically in guard cells of tomato and *Arabidopsis* plants reduces stomatal conductance, decreases transpiration and increases WUE by about 10–20%. It is generally accepted that reducing stomatal conductance (*g*_s_) will inevitably lower the amount of CO_2_ taken up, leading to a lower rate of photosynthesis and decreased biomass production and yield (Lawson and Blatt, 2014; Flexas et al., 2015; Flexas, 2016). Therefore, it has been suggested that it might be possible to improve WUE without negatively affecting photosynthesis by improving CO_2_ mesophyll conductance (*g*_m_) or enhancing the activity of photosynthetic and Calvin cycle enzymes (Sade et al., 2014; Flexas et al., 2015; Simkin et al., 2015; Głowacka et al., 2018). However, tomato and *Arabidopsis* GCHXK plants exhibit reduced stomatal conductance and transpiration with no negative effects on their photosynthesis or growth, indicating that modulation of stomatal aperture is a valid approach for increasing WUE without any drawbacks.

The apparently unexpected effects of reduced stomatal conductance with no restriction on CO_2_ assimilation may indicate that maximal stomatal conductance is not absolutely necessary for the achievement of high rates of photosynthesis. This conclusion is supported by results obtained in a study that followed the speed of changes in the rate of photosynthesis (A) and stomatal conductance (*g*_s_) among several plant species, in response to light changes (McAusland et al., 2016). When various species were transferred from shade (100 µmol m^-2^ s^-1^) to light (1,000 µmol m^-2^ s^-1^), their rates of photosynthesis increased almost immediately and reached 95% saturation within 10–30 min, while their stomatal conductance lagged in most cases and their maximum *g*_s_ levels were reached significantly later (McAusland et al., 2016). These measurements indicate that *g*_s_ does not necessarily limit A and that a high rate of photosynthesis can co-occur with partial stomatal conductance.

Other studies have also suggested that a lower *g*_s_ does not necessarily decrease CO_2_ diffusion and limit A. GTL1 is a transcription factor that controls stomatal development (Yoo et al., 2010). *gtl1* mutants have reduced stomatal density and lower stomatal conductance and transpiration, but nevertheless their CO_2_ assimilation rates are unaffected and their *I*WUE is increased (Yoo et al., 2010). Doheny-Adams et al. (2012) also investigated the impact of epidermal patterning factor mutants with altered stomatal density on *g*_s_, photosynthesis and WUE. Plants that overexpressed *EPF2* showed reduced stomatal density and transpiration, but had greater growth rates and greater biomass (Doheny-Adams et al., 2012). GCHXK plants have lower *g*_s_, but this does not impair their photosynthesis rate or growth, indicating that maximal *g*_s_ is not necessarily required for a plant to achieve high A and that modulation of stomatal aperture may improve WUE.

The observation that maximal stomatal conductance is not absolutely required for high photosynthetic rates highlights the existence of other limitations for high rates of photosynthesis and growth. One such limitation is mesophyll conductance, that is, the diffusion of CO_2_ from the sub-stomatal cavity across the plasma and the chloroplast-membranes to the carboxylation site within the chloroplast. Yet, mesophyll conductance was similar in WT and GCHXK plants (**Figure 5D**) and could not account for the improved performance of GCHXK plants. Another known limitation is the biochemistry of the photosynthetic system, specifically, the capacity of the photosynthetic enzymatic machinery to incorporate CO_2_ (Flexas et al., 2015). Here again, there is no evidence that GCHXK plants possess more efficient photosynthetic capabilities, as the expression of photosynthetic genes is similar in both WT and GCHXK plants (Kelly et al., 2017b) and their *A*/*Ci* curves (**Figure 5G**) are also similar.

Sugar-loading and translocation also might potentially limit photosynthesis and growth. Sugars produced through photosynthesis are loaded into the phloem and exported to sink tissues by pressure flow, but the capability for sugar-loading and translocation might be limited not only by sink strength, but also by the capacity of the phloem. Phloem sugar-loading and translocation are dependent on leaf water potential and the buildup of pressure flow within the phloem (Sevanto, 2014). GCHXK plants have higher leaf water potentials (Kelly et al., 2017b) and it is likely that this accelerates their sugar-loading and translocation. When sugar production exceeds the translocation capacity, sugar accumulates in the leaf (Lu et al., 1997; Ewert et al., 2000; Outlaw and De Vlieghere-He, 2001; Kang et al., 2007) and feedback-inhibits the rate of photosynthesis (Goldschmidt and Huber, 1992; Paul and Foyer, 2001). Improved sugar translocation in GCHXK plants may not only ease sugar feedback-inhibition of photosynthesis, but also boost growth, helping to explain the enhanced growth of GCHXK plants.

Effects similar to those of GCHXK were obtained in tobacco (*Nicotiana tabacum*) plants expressing nicotinamide adenine dinucleotide phosphate (NADP)-malic enzyme (NADP-ME) in their guard cells and vascular companion cells (Müller et al., 2018). Expression of NADP-ME reduced malate content and decreased stomatal aperture, but nevertheless increased the net CO_2_ fixation rate and phloem sugar translocation. That led to less water consumption and more biomass production and, subsequently, improved plant growth and enhanced WUE (Müller et al., 2018). Although no changes in the leaf water potential of the NADP-ME plants were reported, we would expect that lower transpiration due to reduced stomatal aperture would be accompanied by increased leaf water potential, as is the case in GCHXK plants (Kelly et al., 2017b). It is, therefore, likely that, in both cases, increased leaf water potential accelerates phloem sugar translocation and enhances growth.

Unlike the NADP-ME plants, which exhibited smaller stomatal apertures due to lower levels of malate in their guard cells, GCHXK plants exhibit smaller stomatal aperture in response to high sugar levels. During the photoperiod, when sugar production exceeds sugar-translocation capacity, large amounts of sugar are carried by the transpiration stream toward the guard cells and stimulate stomatal closure (Outlaw, 2003; Lawson et al., 2014; Daloso et al., 2016). Expression of HXK in guard cells increases the sensitivity of guard cells to sugars and probably expedites stomatal closure, which, in turn, increases leaf water content and accelerates sugar translocation (Granot and Kelly, 2019). Thus, GCHXK plants exert their stomatal-closure effect primarily at times of day at which sugar levels are high, which may explain why no negative effects on photosynthesis and growth were observed.

### GCHXK and Water Shortage

In this study, we show that GCHXK has two different effects with regard to transpiration: (i) reducing transpiration under well-irrigated and limited-water conditions (**Figures 1**–**5**) and (ii) restraining the reduction of transpiration in situations of water shortage (**Figures 2**, **4**, and **6**). Therefore, GCHXK *Arabidopsis* and tomato plants are less exposed to drought stress due to the fact that their transpiration rates are lower (**Figures 4** and **6**). High stomatal conductance and transpiration levels under well-watered conditions might not impose a problem under normal conditions, but they might be problematic under limited-water conditions. In general, the strategy taken by the GCHXK plants under situations of water shortage seems to be that of drought avoidance (Delzon, 2015), more specifically, *soil water deficit avoidance* (Gilbert and Medina, 2016). The GCHXK plants delay their exposure to drought by draining the soil more slowly (**Figure 6A**). This provides GCHXK plants with the ability to survive longer drought periods while maintaining rather high levels of productivity for a longer time, due to the maintenance of high photosynthesis rates over a longer period of time (**Figures 4**, **6**, **S1**, and **S2**). Soil water-deficit avoidance might be a major advantage in the field, where plants are exposed to episodes of limited water supply. Since total growth depends on the sum of all drought events, the GCHXK plants’ drought avoidance may significantly improve their overall growth.

### Potential Use of GCHXK

It has been suggested that selection for high productivity in crop-development programs has inevitably resulted in the development of cultivars that lose relatively high amounts of water as a side effect of their increased productivity (Richards, 2000; Yoo et al., 2009; Fischer, 2011). This is probably due to the selection of plants with more open stomata, increased stomatal density and/or reduced stomatal sensitivity to environmental signals, all of which lead to greater uptake of CO_2_ from the atmosphere and increased water loss *via* the stomata. It is, therefore, reasonable to assume that many commercial cultivars suffer from wasteful and inefficient water management that can be improved by modifying their stomatal responses.

The GCHXK approach for increasing WUE is comprised of a guard cell-specific promoter (*KST1*ppro), which drives guard cell-specific expression of *Arabidopsis hexokinase1* (*AtHXK1*). Hexokinase is an evolutionarily conserved enzyme that exists in all plant species (tested so far) and the physiological function of *AtHXK1*, in particular, has been found in all plant species tested to date (Jang et al., 1997; Dai et al., 1999; Veramendi et al., 2002; Moore et al., 2003; Cho et al., 2009; Karve et al., 2010; Granot et al., 2013). *KST1*ppro drives guard cell-specific expression in multiple species (Muller-Rober et al., 1998; Plesch et al., 2001; Kelly et al., 2013; Lugassi et al., 2015; Kelly et al., 2017a) and stomatal closure by sugars evolved early in evolution and is conserved across a diverse group of plants (Kottapalli et al., 2018). Taken together, it is likely that the GCHXK method may have similar positive effects in a wide range of species. Indeed, the GCHXK method has been proven effective not only in *Arabidopsis* (Brassicaceae) and tomato (Solanaceae), but also in citrus (Rutaceae) (Lugassi et al., 2015). Therefore, we propose that the GCHXK method may potentially be used in divergent crop species.

## Data Availability Statement

All datasets generated for this study are included in the article/**Supplementary Material**.

## Author Contributions

GK, MM, and DG planned and designed the research. GK and DG wrote the manuscript. AE, GK, BK, and NL performed the experiments. GK, AE, and ZA analyzed the data.

## Funding

This research was supported by The Israel Ministry of Agriculture, Chief Scientist Research Grant 261-0845 and by grant no. IS-4541-12 from Binational Agricultural Research and Development, the United States–Israel Binational Agricultural and Development Fund.

## Conflict of Interest

The authors declare that the research was conducted in the absence of any commercial or financial relationships that could be construed as a potential conflict of interest.

## Abbreviations

GCHXK, guard-cell hexokinase; HXK, hexokinase; *I*WUE, instantaneous water-use efficiency; WUE, water-use efficiency.

## Supplementary Material

Figure S1 Performance of *Arabidopsis* GCHXK plants under drought. Four-week-old WT and GCHXK2 plants were exposed to 13 days of intensifying drought. **(A)** Whole-rosette leaf area of WT (black columns) and GCHXK2 (gray columns) plants at the end of the drought, on day 13. Data points are means of twelve independent biological replicates ± SE. An asterisk denotes a significant difference relative to the WT (*t*-test, *P*< 0.05). **(B)** Representative images of WT and GCHXK2 plants following drought.

Figure S2 Performance of *Arabidopsis* GCHXK plants under mild drought. Two-week-old WT and GCHXK2 plants were exposed to three cycles of drought and re-watering. **(A)** Representative images of whole rosettes of WT and GCHXK2 cut above the ground at the end of the experiment. **(B)** Leaf area of WT (black columns) and GCHXK2 (gray columns) following the drought experiment. Data points are means of ten independent biological replicates ± SE. An asterisk denotes a significant difference relative to the WT (*t*-test, *P*< 0.05).
