# Supplementary figures and images for: Guard-Cell Hexokinase Increases Water-Use Efficiency Under Normal and Drought Conditions
Source: Front Plant Sci. 2019 Nov 19;10:1499. doi: 10.3389/fpls.2019.01499 (PMC6877735; doi:10.3389/fpls.2019.01499)

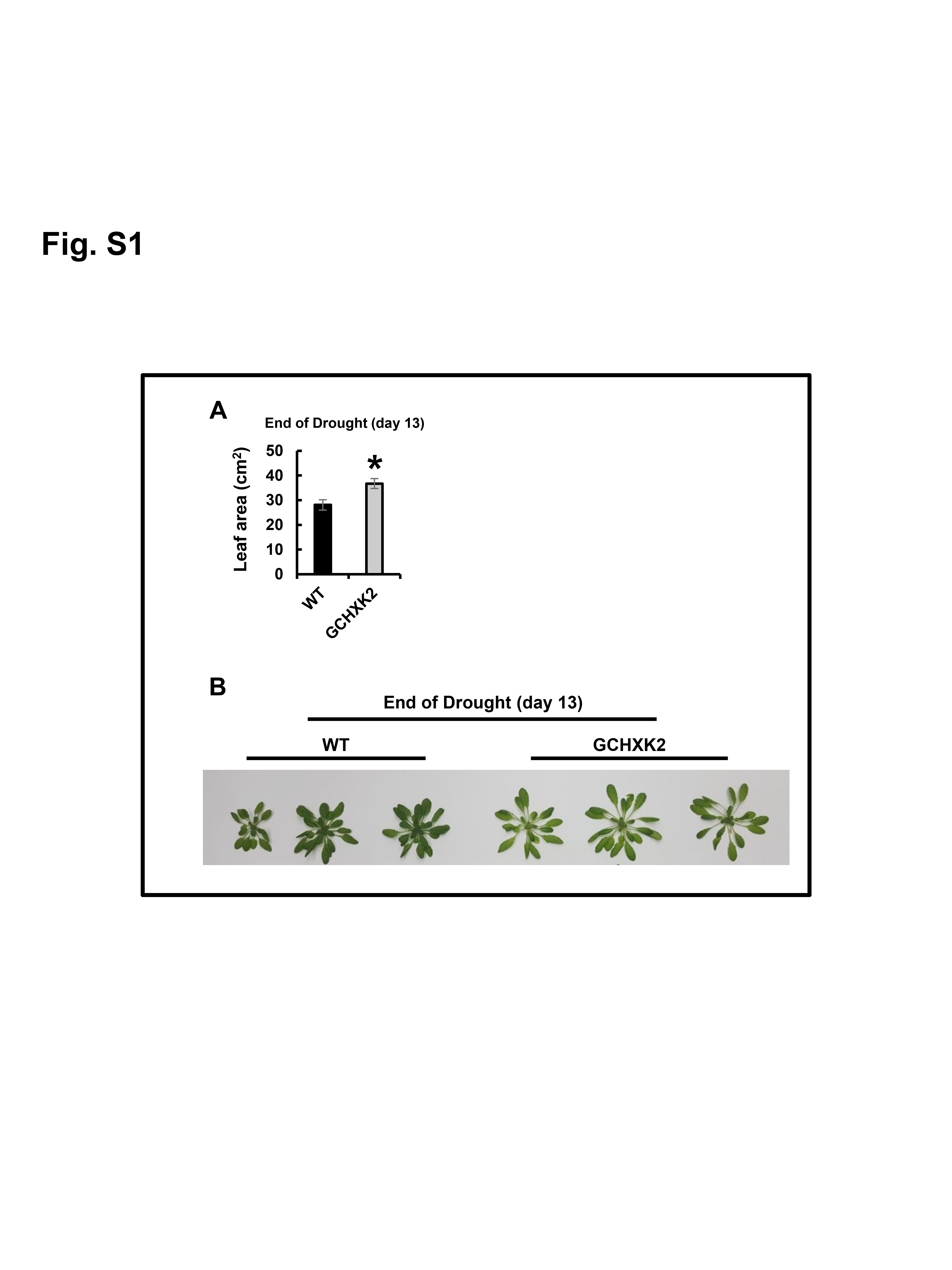

Supplement: Figure S1 — Performance of Arabidopsis GCHXK plants under drought. Four-week-old WT and GCHXK2 plants were exposed to 13 days of intensifying drought. (A) Whole-rosette leaf area of WT (black columns) and GCHXK2 (gray columns) plants at the end of the drought, on day 13. Data points are means of twelve independent biological replicates ± SE. An asterisk denotes a significant difference relative to the WT (t-test, P< 0.05). (B) Representative images of WT and GCHXK2 plants following drought. [file Image_1.jpeg]

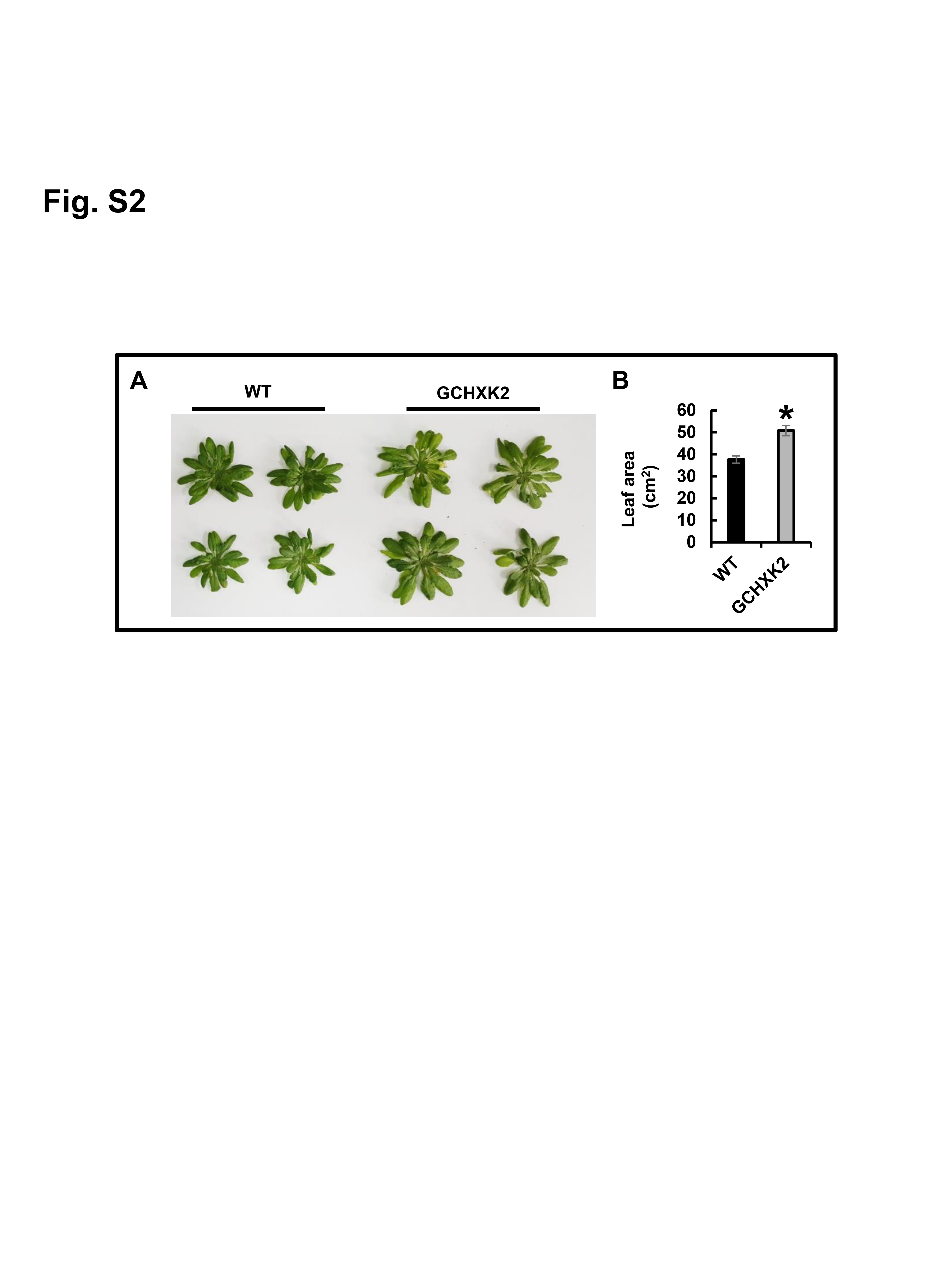

Supplement: Figure S2 — Performance of Arabidopsis GCHXK plants under mild drought. Two-week-old WT and GCHXK2 plants were exposed to three cycles of drought and re-watering. (A) Representative images of whole rosettes of WT and GCHXK2 cut above the ground at the end of the experiment. (B) Leaf area of WT (black columns) and GCHXK2 (gray columns) following the drought experiment. Data points are means of ten independent biological replicates ± SE. An asterisk denotes a significant difference relative to the WT (t-test, P< 0.05). [file Image_2.jpeg]
